# High prevalence of lipoatrophy in pre-pubertal South African children on antiretroviral therapy: a cross-sectional study

**DOI:** 10.1186/1471-2431-12-183

**Published:** 2012-11-23

**Authors:** Steve Innes, Mark F Cotton, Richard Haubrich, Maria M Conradie, Margaret van Niekerk, Clair Edson, Helena Rabie, Sonia Jain, Xiaoying Sun, Ekkehard W Zöllner, Stephen Hough, Sara H Browne

**Affiliations:** 1 Department of Paediatrics, Children’s Infectious Diseases Clinical Research Unit (KID CRU), Tygerberg Children’s Hospital, Stellenbosch University, Cape Town, South Africa; 2 Antiviral Research Centre, University of California, San Diego, USA; 3 Department of Medicine; Division of Endocrinology, Stellenbosch University, Cape Town, South Africa; 4 Department of Dietetics, Stellenbosch University, Cape Town, South Africa; 5 Department of Paediatrics, Stellenbosch University, Cape Town, South Africa; 6 Biostatistics Research Center, Department of Family and Preventive Medicine, University of California, San Diego, USA; 7 Department of Paediatrics; Division of Endocrinology, Stellenbosch University, Cape Town, South Africa; 8 Department of Medicine, Division of Infectious Diseases, University of California, San Diego, USA

## Abstract

**Abstract:**

**Background:**

Despite changes in WHO guidelines, stavudine is still used extensively for treatment of pediatric HIV in the developing world. Lipoatrophy in sub-Saharan African children can be stigmatizing and have far-reaching consequences. The severity and extent of lipoatrophy in pre-pubertal children living in sub-Saharan Africa is unknown.

**Methods:**

In this cross-sectional study, children who were 3-12 years old, on antiretroviral therapy and pre-pubertal were recruited from a Family HIV Clinic in South Africa. Lipoatrophy was identified and graded by consensus between two HIV pediatricians using a standardized grading scale. A professional dietician performed formal dietary assessment and anthropometric measurements of trunk and limb fat. Previous antiretroviral exposures were recorded. In a Dual-Energy X-ray Absorbtiometry (DXA) substudy body composition was determined in 42 participants.

**Results:**

Among 100 recruits, the prevalence of visually obvious lipoatrophy was 36% (95% CI: 27%–45%). Anthropometry and DXA measurements corroborated the clinical diagnosis of lipoatrophy: Both confirmed significant, substantial extremity fat loss in children with visually obvious lipoatrophy, when adjusted for age and sex. Adjusted odds ratio for developing lipoatrophy was 1.9 (95% CI: 1.3 - 2.9) for each additional year of accumulated exposure to standard dose stavudine. Cumulative time on standard dose stavudine was significantly associated with reductions in biceps and triceps skin-fold thickness (p=0.008).

**Conclusions:**

The prevalence of visually obvious lipoatrophy in pre-pubertal South African children on antiretroviral therapy is high. The amount of stavudine that children are exposed to needs review. Resources are needed to enable low-and-middle-income countries to provide suitable pediatric-formulated alternatives to stavudine-based pediatric regimens. The standard stavudine dose for children may need to be reduced. Diagnosis of lipoatrophy at an early stage is important to allow timeous antiretroviral switching to arrest progression and avoid stigmatization. Diagnosis using visual grading requires training and experience, and DXA and comprehensive anthropometry are not commonly available. A simple objective screening tool is needed to identify early lipoatrophy in resource-limited settings where specialized skills and equipment are not available.

## Background

**Figure 1:**
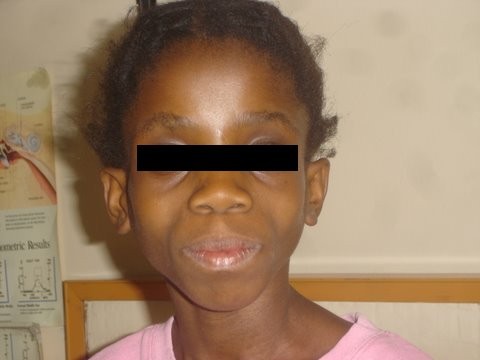
Stigmatizing lipoatrophy in a child – front view.

**Figure 2:**
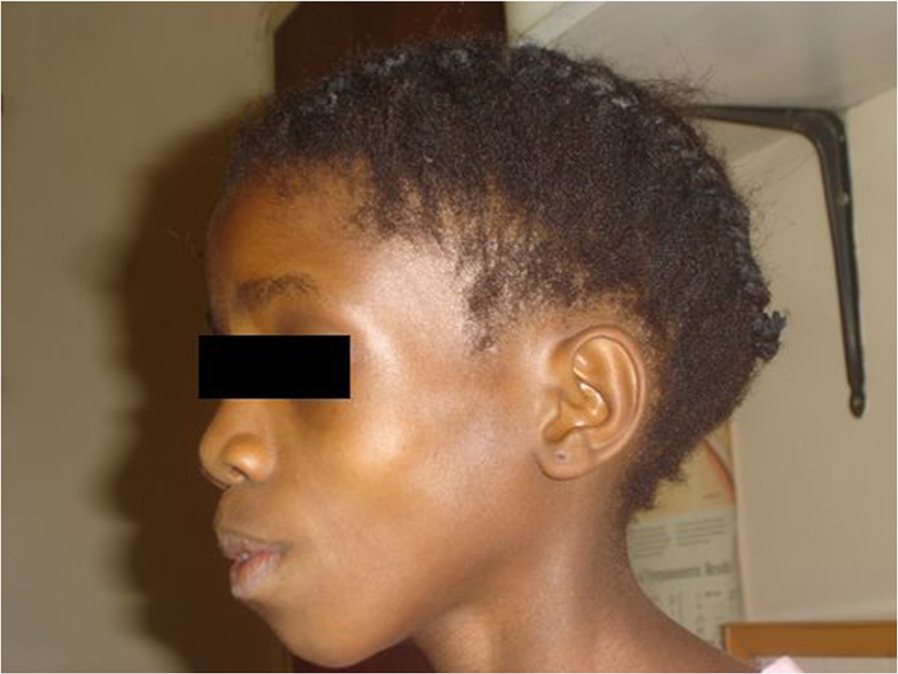
Stigmatizing lipoatrophy in a child – side view.

The introduction of antiretroviral therapy (ART) in sub-Saharan Africa has been life saving. However, long-term ART, particularly nucleoside reverse transcriptase inhibitors, may result in disfiguring loss of subcutaneous fat, termed lipoatrophy (Figures 1 and 2). Lipoatrophy looks very similar to AIDS wasting syndrome, termed “Slims disease” throughout Africa, and may confer the same stigmatization [1]. In contrast to the developed world, stigmatization due to HIV in communal sub-Saharan African cultures may lead to loss of access to communally-held resources, specifically loss of housing, denial of schooling, denial of healthcare, loss of employment or livelihood, secondary stigmatization of family members and physical violence [2,3]. ART-induced lipoatrophy may not be reversible, since lipoatrophy involves apoptosis of adipocytes [4], as opposed to nutritional wasting where adipocyte fat stores shrink but the cell survives. Fear of developing lipoatrophy may cause caregivers to become non-adherent with ART, leading to loss of CD4 cells, subsequent opportunistic infection and possibly death. In multivariate logistic regression modeling, fat distribution abnormalities due to ART were an independent risk factor for subsequent non-adherence in adults [5].

While lipoatrophy has been well described in Europe and the United States of America, there is almost no data on this condition in pre-pubertal children from sub-Saharan Africa, despite over 90% of the 3.4 million HIV-infected children worldwide living in sub-Saharan Africa [6]. In South Africa alone, more than a half million children under 15 years of age are HIV-infected [6] and, despite reductions in vertical transmission, an additional 59,000 new infections in children occur anually [7]. South Africa has the largest antiretroviral treatment program in the world, with an estimated 163,000 infants and children on ART by August 2012 (personal communication – South African National Department of Health), and many more added each year [7]. These children face a lifetime of ART exposure and cumulative ART toxicities, extending into several decades. In addition, the rapid changes related to growth may make them more sensitive than adults to drug-related changes in fat metabolism. The severity and extent of lipoatrophy in pre-pubertal children living in sub-Saharan Africa is unknown [8].

We explored the prevalence and risk factors for lipoatrophy in a group of pre-pubertal South African children on ART. We also correlated the visual diagnosis of lipoatrophy with objective anthropometric measurements of body fat in the whole group, and with Dual Energy X-ray Absorptiometry (DXA) findings in a subgroup.

## Methods

The Family Clinic for HIV at Tygerberg Children’s Hospital is a public sector clinic providing ART to infants and children from the Northern suburbs of Cape Town. In this cross-sectional study, children who were 3–12 years old, on antiretroviral therapy and pre-pubertal were recruited. Pre-pubertal status was determined using Tanner staging. Using review of our electronic health record database, we identified 190 subjects that potentially met inclusion criteria. Of these, 124 attended clinic during the study period and could be approached for screening. A total of 121 provisionally agreed to participate, but 21 did not attend the study visit nor did they respond to attempts at further contact. One hundred subjects were finally recruited. There was no difference in demographic characteristics of the 100 enrolled subjects and the 90 who were not recruited (p>0.20 for age, gender, cumulative time on standard dose stavudine and CD4). Lipoatrophy was identified and graded by consensus between two HIV pediatricians who were experienced in identifying lipoatrophy, using the following lipoatrophy grading scale defined by existing literature [9-11]: 0 – No fat changes; 1 – Possible minor changes, noticeable only on close inspection; 2 - Moderate changes, readily noticeable to an experienced clinician or a close relative who knows the child well; 3 – Major changes, readily noticeable to a casual observer. The sites inspected were face, arms, legs and buttocks. Where the assessment of the two investigators did not concur, the change was graded as the lower score. Lipoatrophy was defined as a score of 2 or 3.

Durations of previous antiretroviral exposures and demographics were recorded from our electronic health record database. HIV RNA and CD4 values were extracted from our central electronic laboratory results server. Doses of antiretroviral drugs followed nationally prescribed protocols. For stavudine this meant a minimum of 1 mg/kg twice daily rounded up to the nearest practical dose. A professional dietician performed anthropometric measurements of trunk and limb fat using a non-stretchable tape-measure (model number F10-02DM, Muratec KDS Corporation, Kyoto, Japan), a high-precision Harpenden® skin-fold caliper (Baty International, West Sussex, United Kingdom), a ShorrBoard® stadiometer (Shorr Productions, Maryland, USA), and a precision weighing scale (model number UC-321, A&D Company, Tokyo, Japan) which was calibrated daily. Measurements included mid upper arm circumference, mid-thigh circumference, chest circumference, waist circumference, hip circumference, biceps skin fold thickness (SFT), triceps SFT, iliac crest SFT, sub-scapular SFT, mid-thigh SFT, height and weight. The following ratios were derived: waist to hip circumference ratio; body mass index; torso to arm SFT ratio ([subscapular + iliac crest SFT] / [biceps + triceps SFT]).

In a DXA substudy, as many DXA scans as logistically possible were performed using a Hologic Discovery (Bedford, Massachusetts, USA) to objectively confirm lipoatrophy. Trunk and limb fat mass, lean mass and fat percentage were measured. Selection was semi-random in that DXA was requested for all recruits; however, as DXA is a rare commodity in the developing world, DXA was not always available. There was no difference in gender, cumulative time on standard dose stavudine or CD4 between the 42 subjects who underwent DXA scanning and the 58 who did not (p>0.50 for all). The children who underwent DXA were marginally younger than those who did not (7.1 versus 8.0 years, p=0.03).

Since very little is known about the normal pediatric population in South Africa, additional normative data was collected from healthy age-, gender- and socioeconomically-matched HIV-uninfected children from the same community who had been enrolled as part of a different study at our research unit [12]. We gathered anthropometry and DXA data from 57 children in order to supplement what is known from published population norms for age-related anthropometric and DXA variables, which are currently only derived from developed country pediatric populations. This local normative data appears in the results tables to assist the reader to gauge the magnitude of the changes found in lipoatrophy-affected children.

In univariate analyses, a t-test was used for parametric data, Wilcoxon rank sum test was used for non-parametric data, and Fisher’s exact test was used for categorical data. Non-parametric continuous data are quoted as median (interquartile range), and parametric continuous data are quoted as mean (standard deviation) or mean (95% confidence interval) where appropriate. Multiple logistic regression was used to study the risk factors associated with visually obvious lipoatrophy, adjusting for age and CD4. Multiple linear regression models were used to compare fat distributions captured by anthropometry and DXA between children with and without lipoatrophy, adjusting for age and sex. Skin-fold thickness data was log-transformed before analysis. All statistical analyses were performed using R version 2.10.0 (Bell Laboratories, New Jersey).

This study was designed in accordance with the guidelines of the International Conference on Harmonization for Good Clinical Practice and with the Declaration of Helsinki (version 2000), and approved and monitored by the Ethics Committee for Human Research of the Stellenbosch University, approval reference number N08/11/349. Written informed consent was obtained from each caregiver prior to participation, and informed assent was obtained from capable children. All patient-related data were stored in a password-secured database under a patient identifying number and kept strictly confidential. This study will directly benefit the community from which the data is drawn by making recommendations about best practice in public sector HIV clinics in South Africa.

## Results

One hundred subjects were recruited. The prevalence of visually obvious lipoatrophy was 36% (95% confidence interval: 27% to 45%). Overall, children with and without lipoatrophy had similar weight-for-age Z-score, height-for-age Z-score, gender distribution, ethnic distribution, WHO clinical stage, viral load and mean CD4 (see Table 1). In children with lipoatrophy, face changes were graded as more severe than limb changes in 11/36; the same as limb changes in 24/36; and less severe than limb changes in 1/36. In all cases, lipoatrophy changes were deemed symmetrical. Twelve of the 36 cases had severe changes (grade 3) similar to the child represented in Figures 1 and 2. Six of the 64 children without lipoatrophy had a maximum lipoatrophy score at any site of grade 1, and 58 had a score of zero at all sites.

**Table 1:** Comparison of HIV-infected children with and without visually obvious lipoatrophy

	**Children with lipoatrophy N=36**	**Children without lipoatrophy N=64**	**Univariate p-value (two-tailed)**	**Multivariate p-value (two-tailed)**^**a**^
Nadir absolute CD4 before ART initiation with standard deviation (SD)	681 (493)	886 (650)	0.29	–
Nadir CD4% before ART initiation (SD)	14% (8%)	19% (8%)	>0.05	–
Maximum WHO clinical stage ever reached: 1 / 2 / 3 / 4	25% / 11% / 39% / 25%	17% / 9% / 46% / 28%	0.78	–
Median age at antiretroviral therapy (ART)	24 (9 – 43)	19 (9 – 37)	0.74	–
initiation, with inter-quartile range (IQR)				
Median age at recruitment (months) (IQR)	89 (71 – 112)	71 (50 – 92)	0.001	0.75
Gender: Male / Female	21 (58%) / 15 (42%)	31 (48%) / 33 (52%)	0.41	–
Mean weight for age Z-score (SD)	−1.1 (1.1)	−0.9 (1.1)	0.39	–
Mean height for age Z-score (SD)	−1.3 (1.2)	−1.4 (1.1)	0.49	–
Mean body mass index Z-score (SD)	−0.62 (1.00)	−0.04 (1.08)	0.008	–
Absolute CD4 at recruitment (SD)	1296 (598)	1223 (631)	0.57	–
CD4% at recruitment (SD)	35% (7%)	32% (9%)	0.03	0.34
Mean current log_10_ viral load (SD)	1.98 (0.45)	2.23 (0.83)	0.10	–
Proportion on 2nd line therapy, defined as switch of ≥2 antiretroviral drugs	8%	6%	0.58	–
Any antiretroviral exposure, median months (IQR)	56 (44 – 75)	43 (25 – 60)	0.002	0.52
Stavudine, median months (IQR)	41 (27 – 48)	30 (7 – 49)	0.02	0.002^b^
Lamivudine, median months (IQR)	52 (41 – 72)	41 (25 – 58)	0.01	0.66
Lopinavir/r, median months (IQR)	26 (0 – 56)	36 (6 – 51)	0.58	0.82
Efavirenz, median^c^ months (IQR)	0 (0 – 44)	0 (0 – 4)	0.003	0.48

^a^ Variables with dashes were not included in the multivariate model.

^b^ Adjusted odds ratio: 1.9 (95% CI: 1.3 - 2.9) for each additional year of accumulated exposure.

^c^ Since fewer than half of the subjects in each group had been exposed to efavirenz, the median efavirenz exposure in both groups was 0 months. Mean efavirenz exposure was 23 months in HIV positive with lipoatrophy versus 7 months in HIV positive without lipoatrophy.

Table 1 reflects that, on univariate analysis, overall time on ART, time on standard dose stavudine, cumulative lamivudine exposure, cumulative efavirenz exposure, and greater age were associated with visually obvious lipoatrophy. Zidovudine and didanosine were not included in the analysis as too few children had been exposed to these drugs. WHO clinical stage did not correlate with the presence or absence of lipoatrophy (p=0.78). A multivariate logistic regression model controlling for age and CD4%, incorporating all antiretroviral exposures found significant in the univariate analysis, identified cumulative time on standard dose stavudine as the predominant risk factor independently associated with lipoatrophy (adjusted odds ratio = 1.9 for each additional year of accumulated exposure to standard dose stavudine; 95% CI: 1.3 to 2.9; p=0.002), while efavirenz and lamivudine were no longer associated. Abacavir exposure was not included in the multivariate model as it was only used for children who had developed a complication of stavudine therapy. Cumulative time on standard dose stavudine was significantly associated with reductions in biceps and triceps SFT (p=0.008). Thirty-five of the 36 children with lipoatrophy and 53 of the 64 children without lipoatrophy had ever been exposed to stavudine, and 51/64 (80%) of children without lipoatrophy were on stavudine-based regimens at recruitment. Table 1 shows the comparative antiretroviral exposures and data from the univariate and multivariate analysis. All but one of the children with lipoatrophy had been exposed to more than 18 months of stavudine therapy. At the time of assessment, 29/36 children with lipoatrophy had been off stavudine for at least six months with no resolution of their symptoms.

Adjusting for age and sex, anthropometrics confirmed significant, substantial extremity fat loss in children with visually obvious lipoatrophy (see Table 2). There were no statistically significant differences in anthropometric measures of fat amount and distribution between children without lipoatrophy and the local HIV-uninfected pediatric population. Biceps SFT, triceps SFT, torso-to-arm SFT ratio and waist-to-hip circumference ratio correlated with maximum lipoatrophy grading score, giving regression coefficients of 0.34 (0.17 to 0.65, p=0.002), 0.31 (0.17 to 0.59, p=0.0006), 3.36 (1.57 to 7.16, p=0.002) and 4.23 (0.37 to 8.10, p=0.03) respectively when adjusted for age and sex.

**Table 2:** Anthropometric measurements in children with and without visually obvious lipoatrophy, adjusted for age and sex

	**Children with lipoatrophy, N=36**	**Children without lipoatrophy, N=64**	**HIV-uninfected local population norms, N=57**	p-value, adjusted for age and sex (two-tailed)*
Biceps skin-fold thickness (SFT), mm, mean with 95% confidence interval (95% CI)	4.2 (3.6 – 4.7)	5.3 (4.9 – 5.7)	5.5 (5.0 – 5.9)	0.002
Triceps SFT, mm, mean (95% CI)	7.1 (6.2 – 7.9)	8.9 (8.3 – 9.6)	8.7 (8.1 – 9.4)	<0.001
Torso-to-arm SFT ratio: mean (95% CI)**	1.05 (0.96 – 1.14)	0.88 (0.81 – 0.94)	0.84 (0.78 – 0.91)	0.002
Waist-to-hip circumference ratio: mean (95% CI)	0.97 (0.96 – 0.99)	0.95 (0.93 – 0.96)	0.91 (0.90 – 0.93)	0.009

* p-value compares HIV-infected children with and without lipoatrophy.

** Torso to arm SFT ratio = (subscapular + iliac crest SFT) **/** (biceps + triceps SFT).

In the DXA substudy, measures of fat amount and distribution confirmed significant, substantial extremity fat loss in children with visually obvious lipoatrophy, when adjusted for age and sex (see Table 3). There were no statistically significant differences in DXA measures of fat amount and distribution between children without lipoatrophy and the local HIV-uninfected pediatric population. Limb fat versus limb lean ratio, limb fat versus total body weight ratio, limb fat versus body mass index ratio and total limb fat (converted to kilograms) correlated with maximum lipoatrophy grading score, giving regression coefficients of −2.24 (−0.86 to −3.62, p=0.003), -0.02 (−0.01 to −0.03, p=0.002), –0.015 (−0.006 to −0.024, p=0.003) and −0.8 (−0. 3 to −1.4 grams, p=0.006) respectively when adjusted for age and sex.

**Table 3:** Dual Energy X-ray Absorptiometry measurements in children with and without visually obvious lipoatrophy

	**Children with lipoatrophy, N=15**	**Children without lipoatrophy, N=27**	**HIV-uninfected local population norms, N=34**	p-value, adjusted for age and sex (two-tailed)*
Limb fat versus limb lean ratio: mean with 95% confidence interval (95% CI)	0.36 (0.25 – 0.46)	0.62 (0.54 – 0.70)	0.63 (0.56 – 0.70)	<0.001
Limb fat versus total body weight ratio: mean (95% CI)	9.9 (8.5 – 11.4)	13.7 (12.7 – 14.8)	14.7 (13.7 – 15.7)	<0.001
Limb fat versus body mass index ratio: mean (95% CI)	0.12 (0.10 – 0.13)	0.15 (0.14 – 0.17)	0.17 (0.16 – 0.19)	0.001
Total limb fat, kg, mean (95% CI)	1.7 (1.4 – 2.1)	2.3 (2.1 – 2.6)	2.7 (2.4 – 2.9)	0.01

* p-value compares HIV-infected children with and without lipoatrophy.

## Discussion

This is the first data from sub-Saharan Africa to show the severity and extent of antiretroviral-related lipoatrophy in children. Visually obvious lipoatrophy was present in a third of pre-pubertal children on ART. Children with visually obvious lipoatrophy had 25-40% less extremity fat (depending on the choice of measurement) than HIV-infected children without lipoatrophy. Children with lipoatrophy were not sicker and did not start ART at a different age, compared to those without lipoatrophy. Since cumulative time on standard dose stavudine is the greatest risk factor for lipoatrophy, it is not surprising that older children, who had amassed greater stavudine exposure, had more lipoatrophy. Other potential confounding effects associated with the difference in age were adjusted for in the multivariate model.

Although DXA and anthropometry may be more precise measures of subcutaneous fat amount and distribution, a visual grading scale was chosen as the primary outcome measure in this study because the greatest danger of lipoatrophy in sub-Saharan Africa stems from stigmatization. Subtle changes in fat distribution that are not visually obvious are less relevant since they are unlikely to result in stigmatization.

As there is evidence of a genetic determinant in lipoatrophy [13], it is important to study lipoatrophy specifically in sub-Saharan African populations. Our data suggest that the prevalence of visually obvious lipoatrophy in pre-pubertal South African children on ART is higher than the prevalence among most non-African cohorts (typically 10 to 20%) [9,11,14,15]. Earlier studies from the developed world have included immigrant children from sub-Saharan Africa living in Paris [14], London [16] and Brussels [15], and found a lipoatrophy prevalence of 11%, 8% and 20% respectively. In the largest and most comprehensive study to date, Alam et al. [17] found a 28% prevalence in a cross-sectional study of 426 European children, including 85 pubertal and 154 post-pubertal children and 107 black children. The authors of that study noted that their prevalence was significantly higher than previous pediatric studies, in line with our data. Since survival rates are high in medication-adherent HIV-infected children, and established lipoatrophy changes are largely irreversible, prevalence can be expected to increase progressively if incidence remains constant. Alam et al. found that Caucasian rather than African ethnicity was a risk factor for lipoatrophy. However, none of our recruits were Caucasian and our pre-pubertal South African group had a higher prevalence than Alam’s cohort. This is most likely due to higher rates of stavudine exposure in our context. The magnitude of this difference suggests that data from immigrant African populations in Europe cannot be extrapolated to populations living in sub-Saharan Africa and emphasizes how crucial it is to study sub-Saharan populations directly, as >90% of HIV-infected children live in these regions.

Ten percent of the cohort reported by Alam et al. was currently exposed to stavudine [17], compared to 62% of ours. While Alam et al. found an association between lipoatrophy and current stavudine exposure, our results go one step further to show that the risk of lipoatrophy increases progressively as exposure to stavudine accumulates. This finding is in line with that of the Asian cohort reported by Aurpibul et al., which found an increasing prevalence of fat distribution abnormalities as cumulative exposure to ART increased [11]. This is significant since Asian cohorts have similar conditions to sub-Saharan Africa in that malnutrition is common, access to ART for children is incomplete, and stavudine has been the most widely used first-line antiretroviral agent.

The PACT 1045 study [18] compared morphologic changes using DXA in HIV-positive children compared to HIV-negative matched controls in a cross-sectional study. DXA-measured total body and limb fat were lower in the HIV-positive subjects than in the HIV-negative group in models adjusted for race, disease stage, weight, height and Tanner stage. Among HIV-infected subjects, there was no difference in limb fat between subjects who used non-nucleoside reverse transcriptase inhibitors versus protease inhibitors. Duration and use of nucleoside reverse transcriptase inhibitors (including stavudine) were not evaluated in that study.

Objective anthropometric and DXA measurements confirmed the clinical assessment of visually obvious lipoatrophy. Thus, diagnosis of lipoatrophy in African children by skilled visual assessment is reliable and, whilst it requires specific training and experience, no additional investigations are needed in a developing-country context. In our context, specific training to recognize lipoatrophy in children typically includes didactic training followed by supervised practice in a clinic setting over a period of time until competence is reached to the satisfaction of the trainer. However, basic competence might be gained through review of an array of photographs of mild to moderate lipoatrophy at varying ages, combined with diligent vigilance during clinical practice. The authors have not studied the adequacy of photograph-based training.

The prevalence of visually obvious lipoatrophy in pre-pubertal South African children on ART is higher than previously anticipated, affecting a third of children on ART. This is most likely due to more extensive use of stavudine than elsewhere. Whilst ART is life-saving for HIV-infected children, surveillance and early diagnosis of lipoatrophy with appropriate drug-switches is critical. The use of agents associated with potentially stigmatizing face and limb changes is undesirable. The antiretroviral agent most commonly associated with lipoatrophy in adults is stavudine [19] and our findings substantiate this in the pre-pubertal pediatric South African population. The 2010 World Health Organization (WHO) antiretroviral guidelines advise that stavudine should be phased out where possible. However, stavudine remains within the nationally recommended pediatric first-line ART guidelines for numerous developing countries. In South Africa, stavudine was the first choice nucleoside reverse transcriptase inhibitor for children, together with lamivudine, from the beginning of the ART access program in 2004 until 2010, when it was replaced with abacavir. Current South African guidelines state that children taking stavudine should continue unless side-effects develop. Thus the majority of children treated for HIV in South Africa remain on stavudine. The effect of stavudine in causing lipoatrophy appears to be strongly dose-related [20-23]. The current standard pediatric dose of stavudine was determined by extrapolation from the pharmacokinetic parameters of the adult dose of 40 mg twice daily using data from pediatric pharmacokinetic studies [24,25]. Those studies showed that an oral dose of 1 mg/kg/dose twice daily in children under 30 kg results in plasma exposure similar to that of an adult over 60 kg taking 40 mg twice daily; and that an oral dose of 0.5 mg/kg/dose twice daily in children results in plasma exposure similar to that of an adult over 60 kg taking 20 mg twice daily. In 2007 an influential review by Hill et al. of evidence accumulated over the previous 15 years suggested that stavudine given at the dose of 20 or 30 mg twice daily leads to a significantly lower rate of lipoatrophy and of other mitochondrial adverse effects while maintaining excellent antiviral efficacy [20]. In response, the World Health Organization advised that the recommended adult dose be lowered from 40 to 30 mg BD [26]. The children’s dose, however, has not yet been reduced. Consequently children on stavudine continue to be exposed to a disproportionately high dose, which may result in more rapid accumulation of metabolic adverse effects than adults on the reduced dose.

Lipoatrophy was previously thought to be uncommon in children on stavudine. For that reason, the global transition away from stavudine-based regimens has focused on adult services, while children on stavudine-based regimens have not received equal attention. Stavudine remains the most commonly used pediatric antiretroviral in sub-Saharan Africa. The Clinton Health Access Initiative and UNITAID have worked since 2002 to improve antiretroviral drug supply chains for adult formulations in sub-Saharan Africa by facilitating procurement and providing funding for purchases where necessary. Similar efforts are critical to the successful transition away from stavudine-based regimens for children.

Our study has the following limitations: we selected subjects for enrollment based on meeting eligibility requirements and having a routine clinic appointment during the study-screening period. This could represent a biased subset of all available subjects in our clinic population, but there is no reason to think that temporal factors (when a subject had a clinic visit) would be related to the study outcome of lipoatrophy. The relatively high rate of drop-out from study screening to the actual study visit could also bias the sample, but basic demographics were not different and logistic difficulties, due to extreme poverty, often interfere with clinic attendance; patients’ arrival for appointments is typically erratic. Considering these obstacles, a sample size of 100 children is a significant achievement. The subsets of recruits exposed to zidovudine and other nucleoside reverse transcriptase inhibitors were small, and did not allow comment on their relationship to lipoatrophy. The two clinicians who performed the visual assessment could not be blinded to the children’s ART status, which may have biased their assessment. However, since the children were well-known, the clinicians had a longitudinal perspective on which to base their assessment. DXA was requested for all recruits; however, as DXA is a rare commodity in the developing world, DXA was not always available. The possibility of selection bias in the DXA substudy cannot be excluded.

As with the cohort reported by Alam et al., we are collecting longitudinal data on our cohort, which will allow calculation of incidence and will document the degree and rate of regression after antiretroviral switching.

## Conclusions

The prevalence of visually obvious lipoatrophy in pre-pubertal South African children on antiretroviral therapy is high, and is likely to continue rising as stavudine continues to be used extensively at an unnecessarily high dose. The amount of stavudine that children are exposed to needs review. Resources are needed to enable low- and-middle-income countries to provide suitable pediatric-formulated alternatives to stavudine-based pediatric regimens. The standard stavudine dose for children may need to be reduced. Diagnosis of lipoatrophy at an early stage is important to allow timeous antiretroviral switching to arrest progression and avoid stigmatization. Diagnosis using visual grading requires training and experience, and DXA and comprehensive anthropometry are not commonly available. A simple objective screening tool is needed to identify early lipoatrophy in resource-limited settings where specialized skills and equipment are not available.

## Consent

Both photos (Figures 1 and 2) have been previously printed in Innes S, et. al [27]. They are reprinted here with permission from the *South African Journal of HIV Medicine*. Consent for publication of both photos was obtained from the relevant legal guardian.

Part of this data has been presented at the Conference on Retroviruses and Opportunistic Infections (March 5^th^ – 8^th^, 2012, Seattle, WA).

## Competing interest

The authors have no conflict of interest to declare.

## Authors’ contributions

SI, MFC, RH and SHB conceived and planned the study. SI recruited and performed the study, planned the analysis and drafted the manuscript. MFC, RH and SHB supervised the analysis and participated in drafting the manuscript. MMC performed the DXA scans and reviewed the manuscript. MVN performed the anthropometry and diet assessments and reviewed the manuscript. SI and CE performed the visual assessments together and CE reviewed the manuscript. HR contributed to study design, supported recruitment and approved the final manuscript. SJ and XS performed the statistical analysis and participated in manuscript drafting. SH gave intellectual input to the interpretation of DXA analysis and to manuscript drafting. EZ contributed to interpretation of analysis and to manuscript drafting. All authors read and approved the final manuscript.

## Pre-publication history

The pre-publication history for this paper can be accessed here:

http://www.biomedcentral.com/1471-2431/12/183/prepub
